# Response to commentary on “Ultra-rare severe kidney dysplasia mimicking salt-wasting tubulopathy associated with *TFCP2L1* gene variants”

**DOI:** 10.1007/s00467-025-06948-2

**Published:** 2025-09-11

**Authors:** Manuel Vaqueiro Graña, Sara Gómez-Conde, Leire Madariaga

**Affiliations:** 1 Department of Pediatric Nephrology, Vall D’Hebron University Hospital, Barcelona, Spain; 2 Department of Pediatrics, University of the Basque Country, Biobizkaia Health Research Institute, CIBERDEM/CIBERER/Endo-ERN, Barakaldo, Spain; 3 Department of Pediatric Nephrology, Biobizkaia Health Research Institute, University of the Basque Country, CIBERDEM/CIBERER/Endo-ERN, Cruces University Hospital, Barakaldo, Spain

Dear Editors,

We thank Khan et al. for their comments [1] on our recent manuscript “Ultra-rare severe kidney dysplasia mimicking salt-wasting tubulopathy associated with *TFCP2L1* gene variants” [2].

We agree that additional functional studies would help to support the pathogenicity of the identified variant. However, functional studies involving *TFCP2L1* alterations have already been reported [3], and our aim was to present a clinically similar case to further expand the phenotypic spectrum. Therefore, we state that this case provides further evidence of the association between *TFCP2L1* alterations and kidney dysplasia with severe salt-wasting. In addition, although we state that, following the ACMG/AMP criteria, the reported variant is classified as probably pathogenic, these guidelines are intended for use in genes with a well-established association with disease.

Regarding the genetic differential diagnosis, in the initial molecular analyses, we used a comprehensive gene panel targeting genes known to be associated with salt-wasting phenotypes, as this was the patient’s most striking clinical feature. When no causative variant was identified, we proceeded with whole-exome sequencing, examining genes associated with the broader phenotype, including nephrocalcinosis and kidney dysplasia.

Finally, although no separate method for structural variant analysis was used, copy number variants were assessed as part of the whole exome sequencing pipeline using the Franklin by Genoox interpretation platform. Given the identification of a homozygous *TFCP2L1* variant in a patient with a phenotype similar to that previously described [3], we inferred a likely causal relationship and did not pursue further molecular analyses.

## Data Availability

The data presented in this study are from a single individual patient. These data are not publicly available to protect patient confidentiality and comply with ethical standards.
